# Efficiency Analysis as a Tool for Revealing Best Practices and Innovations: The Case of the Sheep Meat Sector in Europe

**DOI:** 10.3390/ani11113242

**Published:** 2021-11-12

**Authors:** Alexandros Theodoridis, Sotiria Vouraki, Emmanuel Morin, Leticia Riaguas Rupérez, Carol Davis, Georgios Arsenos

**Affiliations:** 1 Laboratory of Animal Production Economics, School of Veterinary Medicine, Faculty of Health Sciences, Aristotle University, 54124 Thessaloniki, Greece; 2 Laboratory of Animal Husbandry, School of Veterinary Medicine, Faculty of Health Sciences, Aristotle University, 54124 Thessaloniki, Greece; svouraki@vet.auth.gr (S.V.); arsenosg@vet.auth.gr (G.A.); 3 Institut de l’Élevage, CS 52637, 31321 Castanet Tolosan, France; emmanuel.morin@idele.fr; 4 Oviaragón Pastores Cooperative Group, 50014 Zaragoza, Spain; lriaguas@oviaragon.com; 5 Agriculture and Horticulture Development Board, Kenilworth, Warwickshire CV8 2TL, UK; Carol.Davis@ahdb.org.uk

**Keywords:** sheep farms, efficiency, best practices, innovations, economic performance

## Abstract

**Simple Summary:**

The European sheep meat sector faces technical, market and financial challenges that threaten its economic performance and overall sustainability. At the same time, the sector is characterized by poor and slow adoption of innovations that could help towards facing these challenges. In this study, the technical efficiency of extensive, semi-intensive and intensive sheep meat farms in France, Spain and the UK was explored to reveal the profile of the most efficient ones and identify the best practices and innovations that these farms apply. The most efficient sheep meat farms reared large flocks, used available infrastructure at full capacity and managed human labor in a rational way. These best farms emphasized feeding and grazing innovations, marketing strategies, breeding programs and use of digital technologies. The uptake of such practices and innovations by farms of similar production systems could help to increase the productivity and economic performance of the sheep meat sector.

**Abstract:**

The slow adoption of innovations is a key challenge that the European sheep sector faces for its sustainability. The future of the sector lies on the adoption of best practices, modern technologies and innovations that can improve its resilience and mitigate its dependence on public support. In this study, the concept of technical efficiency was used to reveal the most efficient sheep meat farms and to identify the best practices and farm innovations that could potentially be adopted by other farms of similar production systems. Data Envelopment Analysis was applied to farm accounting data from 458 sheep meat farms of intensive, semi-intensive and extensive systems from France, Spain and the UK, and the structural and economic characteristics of the most efficient farms were analyzed. These best farmers were indicated through a survey, which was conducted within the Innovation for Sustainable Sheep and Goat Production in the Europe (iSAGE) Horizon 2020 project, the management and production practices and innovations that improve their economic performance and make them better than their peers.

## 1. Introduction

Sheep farming is a traditional livestock sector of economic, social, cultural and environmental importance, especially for the economically vulnerable regions of Europe. Sheep farming offers income and employment opportunities in disadvantaged, mountainous and marginal areas where rural economy is poorly diversified [1,2,3], produces high-quality traditional products [4], provides ecosystem and non-market services (biodiversity conservation, flood prevention, water purification, etc.; [5]), preserves social cohesion in rural areas and supports local cultural heritage [6]. There are 85 million sheep in Europe reared on approximately 850,000 farms under diverse production systems; the main producing countries are Greece, Spain, France, Romania and the United Kingdom [7]. 

Over the last years, though, the European small ruminant sector is experiencing economic and structural difficulties, mainly involving a consistent decrease in livestock numbers, following partial decoupling of direct payments from production, changes in the standards of living in rural areas and market liberalization, resulting in a need for innovative, modern and highly competitive farms [8,9]. 

Meat production constitutes the most important activity of the small ruminant sector, accounting for about EUR 5.6 billion, i.e., 6% of the total value of meat production in the European Union (EU) [10]. According to the latest EU statistics, 688 thousand tonnes of sheep and goat meat were produced in 2019, the vast majority (90%) of which was sheep meat [11]. The main sheep meat-producing countries in Europe are the United Kingdom (37% of the EU-28 total production in 2019, when the UK was a member of EU), Spain (about 17% of the total) and France (11%).

However, the sheep meat sector in the EU is characterized by a decrease in total production, severe fluctuations in producer’s price (varying from 4.6 EUR/Kg to 6.4 EUR/Kg during the 2018–2019 period) and high volume of imports from non-EU countries, mainly New Zealand and Australia; self-sufficiency for sheep meat is currently at 80% (EU sheep and goat market dashboard). Moreover, the sector faces significant technical, market and financial challenges, which pose extra threats to its resilience and sustainability. Such challenges include the poor structure of the sector (small farm size, ageing of farmers population and lack of new entrants), depopulation of rural areas, low profitability compared with other agricultural enterprises, high subsidy dependency, farmers’ reluctance to modify farming practices, poor management training and lack of innovation culture across farming communities [12,13,14,15,16]. At the same time, the sheep meat market has been facing increasing competition, highlighting the need to improve the competitiveness of meat production in the EU [17]. In this environment of growing liberalization and competition, it is essential for the European sheep sector to improve its performance and increase its productivity.

High and sustainable economic performance of sheep farms is associated with improved production techniques and effective management practices. In this regard, the identification and adoption of best farm practices and innovations are considered strategic priorities towards enhancing and strengthening the productivity and competitiveness of the sheep sector [15,16,18,19], which is characterized by low innovation adoption rates [13,19]. For this reason, the European Commission, following the recommendations of the Sheepmeat Forum, supports research initiatives for innovative production methods and technologies and highlights the importance of identifying and exchanging the best practices between Member States, both in terms of farming and product marketing [14]. The resilience and the economic sustainability of sheep meat farms in Europe would benefit from the identification and implementation of best practices and innovations to support farm activities and to ensure a satisfactory income.

A practical tool for identifying the best practices, innovations and management strategies to improve the economic performance and sustainability of farms is Technical Efficiency (TE) analysis. The concept of TE is related to the producer’s management capacity and involves the “best” use of scarce resources, which is the foundation of economic performance [20,21,22]. TE analysis allows the measurement of the differences in the level of managerial skills among farmers and indicates the best-practicing farms that utilize the existing production technology efficiently, benchmarking the performance of the rest against the top-performing farms.

This study aspires to contribute to the debate about the development and uptake of innovative solutions for the improvement of productivity in the European sheep meat sector. In particular, the paper explores how the efficiency analysis could serve as a useful decision tool for defining strategies and policies that would induce European sheep farmers to become more innovative and consequently more productive. For this reason, based on farm accounting data from 458 sheep meat farms from France, Spain and the UK, the differences in the level of efficiency are investigated and the best-observed management and production practices that are applied in the most efficient sheep meat farms are revealed. A comparative technical and financial analysis is conducted between the efficient and the inefficient farms for each production system, providing insights regarding the structure and the profile of the best farms. Moreover, the difference between the actual and potential performance of each farm type is estimated, indicating the productive capacity of the sector. 

To our knowledge, this is the first study that uses efficiency analysis to increase knowledge on best practices that could be potentially introduced to sheep farms as innovations. Regarding the European small ruminant sector, the studies on efficiency are limited in the estimation of the level of technical efficiency of the farms and the detection of the sociodemographic and environmental factors that explain efficiency differentials (indicatively see [2,23,24,25,26,27,28,29]). Moreover, all of these studies are focused on the dairy sheep sector; only Perez et al. [23] estimated the efficiency level on the sheep meat sector, without, however, providing insights regarding the structural and economic characteristics of the efficient farms.

## 2. Materials and Methods

### 2.1. Theoretical Model of Data Envelopment Analysis—DEA

The economic literature provides a variety of methods to estimate TE of production units, of which the most celebrated is Data Envelopment Analysis (DEA). DEA has its origins in the seminal work by Charnes et al. [30] and measures TE estimators as optimal solutions to mathematical programming problems. It is a non-parametric, mathematical programming-based technique, which has been extensively used for defining efficiency frontiers and for estimating the level of TE of decision-making units (DMUs). Each unit consumes varying amounts of different inputs to produce different outputs and the level of efficiency is measured relative to the highest observed performance. In general, DEA uses a set of production units in the sample to construct an efficiency frontier consisting of all possible linear combinations of efficient production units. This efficiency frontier is developed through the optimization of the weighted output/input ratio of each production unit, subject to the condition that this ratio for each unit in the sample can equal, but never exceed, unity [31]. Consequently, the efficient units lie by definition on the frontier (TE = 1), whereas the inefficiency of the units that are not on the frontier is indicated in direct proportion to their distance from the frontier (0 ≤ TE < 1). In the present study, DEA is implemented in the sampled sheep farms (DMUs) from France, Spain and the UK to identify the most efficient ones.

Assuming that there are *n* DMUs, each producing a single output by using *m* different inputs and the *i*th DMU produces y_i_ units of output using x_ki_ units of the *k*th inputs, the variable returns to scale (VRS) output-oriented model for the *i*th DMU is expressed as follows:

(1) $$\underset{\theta_{i}{,\lambda }_{j}}{\mathrm{Max}}\theta_{i}$$

subject to

(2) $$\overset{n}{\underset{j=1}{\sum }}\lambda_{j}y_{j}{-\theta }_{i}y_{i}\geq 0\;\overset{n}{\underset{j=1}{\sum }}\lambda_{j}x_{\mathrm{kj}}\leq x_{\mathrm{ki}}\;\overset{n}{\underset{j=1}{\sum }}\lambda_{j}=1\;\lambda_{j}\geq 0;$$

k = 1, …, m inputs; j = 1, …, n DMUs;

where $\theta_{i}$ is the proportional increase in output possible for the *i*th DMU and $\lambda_{j}$ are intensity variables, indicating at what intensity a particular activity may be employed in production. 

The single output-oriented DEA model maximizes the proportional increase in output while remaining within the production possibility set. In this empirical investigation, the output-oriented DEA model was considered the most appropriate to implement in comparison with the input-oriented model [32], because sheep farming is a sector that utilizes resources that would otherwise go unexploited, such as degraded grazing land. The *i*th farm is efficient, which means that the unit lies on the frontier when $\theta_{i}{=1,\;\lambda }_{i}=1$, and $\lambda_{j}=0$ for j≠i. The frontier level of production for the *i*th farm, denoted by $y_{i}^{*}$, is given by

(3) $$y_{i}^{*}=\overset{n}{\underset{j=1}{\sum }}\lambda_{j}y_{j}{=\theta }_{i}y_{i}$$

The output-oriented measure of technical efficiency of the *i*th farm unit, denoted by TE_i_, can be estimated by

(4) $${\mathrm{TE}}_{i}=\frac{y_{i}}{y_{i}^{*}}=\frac{1}{\theta }$$

Comprehensive reviews and extensions of the DEA model can be found in Kumbhakar and Lovell [33], Coelli [34], Cooper et al. [35] and Coelli et al. [22]. 

### 2.2. Empirical Model of DEA

The DEA application in this study was based on technical and economic data collected in 2015 from (i) 241 extensive sheep meat farms and (ii) 61 intensive sheep farms in France, (iii) 37 semi-intensive sheep meat farms in Spain and (iv) 119 extensive sheep meat farms in the UK (Spreadsheets S1–S4). The farm types were defined on the basis of the farm typology developed in iSAGE (Innovation for Sustainable Sheep and Goat Production in Europe) HORIZON 2020 Project (https://www.isage.eu/; accessed on 29 October 2021) [36]. The data were provided by research and breeding organizations, namely by the French Livestock farms network “INOSYS Réseauxd’élevage” in France, Oviaragon in Spain, the National Sheep Association (NSA) and the Agriculture and Horticulture Development Board (AHDB) in UK. DEA was applied for each farm type separately; thus, in total, 4 DEA models were estimated. The application of DEA involves the identification and measurement of relevant inputs and outputs, which are common in all production units. In the specification chosen in this study, the relevant inputs used were: (1) flock size measured by the number of ewes, (2) total labor (including family and hired workers) measured in hours, (3) variable costs in EUR and (4) fixed capital costs in EUR. Gross revenue, including subsidies and compensations, measured in value terms (EUR) was selected as the output variable.

### 2.3. Best Observed Practices in Efficient Farms

The next step in the methodological approach involved the identification of the management and production practices (e.g., use of antibiotic alternatives in feeding, automatic animal handling, training on breeding programs, on-farm data collection linked to animal ID etc.) that the relatively efficient farms, estimated through DEA, implement at full potential (or in a better way than the relatively inefficient farms). In order to indicate the practices that render these farms more efficient, a template of innovations and best observed practices was created (Template S1). The experts that supervise and consult these farms (such as veterinarians, animal husbandry experts, agronomists) used this template to report their feedback. 

The categories of best practices were based on information gathered at workshops with 18 sheep and goat industry partners in Thessaloniki (Greece) and Zaragoza (Spain) in 2016, and an online survey that took place in the context of the iSAGE project. After collecting this information on best practices and innovations from the workshops and the survey, the initial list was organized by grouping those innovations in the following major categories and sub-categories: 

(i) Farm Management, which included 1. Feeding; 2. Health; 3. Reproduction; 4. Breeding; 5. Human resource organizations. 

(ii) Farm Technology, including 1. Information and Training and 2. Gadgets/Apps. 

(iii) Product processing and marketing, including 1. Product processing and 2. Product Marketing. 

The feedback was recorded in the template for all farms in France and Spain, but not for the UK, where there was no accessibility to detailed information about applied farm practices. Then, it was processed and analyzed to extract valuable conclusions regarding the best observed practices in the sheep meat sector.

## 3. Results and Discussion

The variable returns to scale (VRS) output-oriented DEA model was applied using the DEAFrontier program [37] and the frequency distribution of the technical efficiency estimates obtained are presented in Table 1. The results indicate that there are substantial technical inefficiencies in the utilization of the existing production technology in all farm types and countries. The TE score of the French extensive and intensive farms varied from 31.3% and 57.6%, respectively, to a high of 100%, which are the efficient. Many farms (25 extensive and 22 intensive farms, accounting for 10.37% and 36.1%, respectively, of the total sample) were allocated in the fully efficient group, whereas 66 extensive farms and only 1 intensive farm exhibited TE score less than 60%. This result verifies the fact that intensive farms are more homogenous, operating under a standardized pattern that it is not easily diversified. The TE score of 99 extensive (41.08%) and 18 intensive farms (29.5%) was between 60% and 79%, whereas 51 extensive (21.17%) and 20 intensive farms (32.8%) operated relatively close to the DEA frontier, exhibiting TE between 80% and 99%. The mean TE of the 241 extensive and 61 intensive farms was 70.9% and 87.3%, respectively, indicating that given the level of inputs, the average French meat farm could increase its production if it was operating efficiently. The analytical results, which contain not only the proportionate increase that is allowed by the production possibility set, (1-θ), but also any further change associated with no zero slacks [35], suggest that an approximately 37% and 13.6% increase of the production value for the extensive and the intensive farms, respectively, is possible, provided that the farmers optimize the management of their farms, adopting best practices (see the change in revenues of the average farm presented in the last column of Table 2). 

The TE score of the Spanish semi-intensive farms varied from 57% to a high of 100% and 14 farms (37.83% of the total sample) were allocated in the fully efficient group, whereas only 2 farms exhibited TE score less than 60%; 6 farms (16.22%) had TE between 60 and 79 percent and 15 farms (40.54%) operated relatively close to the DEA frontier, exhibiting technical efficiency between 80% and 99%. The mean TE of the 37 Spanish farms was 87.4%, indicating that given the level of inputs the average semi-intensive meat farm in Spain could increase its production if it was operating efficiently. The analytical results suggest that an approximately 13% increase of the gross revenues is possible (last column of Table 2), using the same inputs, provided that the farms adopt the best observed practice. According to Perez et al. [23], where the authors applied an econometric approach for the estimation of TE on 49 extensive sheep meat farms, the mean TE score was 66%. The same score was found from Toro-Mujica et al. [25], who also estimated a Cobb–Douglas function for the measurement of efficiency in 31 organic dairy farms. 

The TE score of the extensive farms in the UK varied from 31% to a high of 100%. Among those, 22 farms, which is the 18.5% of the total sample, were allocated in the fully efficient group, whereas 18 farms exhibited a TE score less than 60%, 52 farms (43.7%) had TE between 60% and 79% and 27 farms (22.7%) operated relatively close to the DEA frontier, exhibiting TE between 80% and 99%. The mean TE of the 119 UK farms was 77.3%, whereas the analytical results suggest that an approximately 23% increase in the production value is possible (last column of Table 2), given the level of inputs, provided that the farms allocate the resources rationally. The presence of TE in all farm types indicates that farmers are relatively insufficient in the utilization of the entrepreneurial factor, a factor that has a great impact on the economic performance of a production unit.

The output-augmenting orientation DEA approach identifies efficiency targets (possible increases in output) while holding the inputs constant [38]. The results of efficiency improvement projection based on the VRS DEA model for all farm types are presented in Table 2. The last column of Table 2 represents the efficient target (DEA projection of the optimal output value) of the farms under evaluation. If the sample farms achieved these targets, they would be operating on the efficient frontier.

The French extensive farms could increase their output if they efficiently utilized the existing production technology, from EUR 81,624 to 111,964, which is an increase of 37.2%, indicating that their profitability and competitiveness could be improved substantially. The average levels of the actual and the efficient frontier outputs of the farms are also presented based on their size in number of ewes. Relatively small farms that rear less than 350 ewes could, on average, increase their output by 51.2% (from EUR 37,751 to 57,088), medium-sized farms (350–450 ewes) by 53.7% (EUR from 63,741 to 97,980) and large-sized farms (more than 450 ewes) by 30.6% (from EUR 118,818 to 155,235). These findings imply that large-sized farms are positively associated with TE and that the sheep farms could increase their production value, and consequently their productivity, by adjusting to an optimal size, considering the labor and land constraints.

The French intensive farms could increase their output, if they optimally utilized the existing technology of production, from EUR 91,582 to 104,035, which is an increase of 13.6%. As expected, the adjustment here is lower than in the extensive system, since intensive farms exhibit higher level of efficiency. Small intensive farms that rear less than 350 ewes could, on average, increase their output by 7.16%, medium-sized farms (350–450 ewes) by 23.12% and large-sized farms (more than 450 ewes) by 10.47%. 

The Spanish semi-intensive farms could increase their output by approximately 13%, an adjustment similar to that in the French intensive farms. Small farms (<600 ewes) could, on average, increase their output by 8.8%, medium-sized farms (600–1000 ewes) by 23.6% and large-sized farms (more than 1000 ewes) by 9.3%. These findings imply that small-sized farms are positively associated with TE and that the sheep farms could increase their production value, and consequently their productivity, by adjusting to an optimal size. This finding also implies that in Spain there is much room for improvement, mainly for the medium-sized farms. 

The UK extensive farms could increase their output by 28.1% if they were valorizing fully the existing technology available in terms of production possibilities, a finding similar to that in the French extensive farms. Small-sized farms (<400 ewes) could, on average, increase their output by 32.8%, medium-sized farms (400–900 ewes) by 37.22% and large-sized farms (more than 900 ewes) by 21.3%, a finding which implies that large-sized farms in the UK are positively associated with TE.

The sample farms were categorized into two efficiency groups that include the relatively inefficient and the efficient farms, respectively. The composition of gross revenue per ewe for each efficiency group and for the average farm was computed and the results are presented in Table 3. Results in Table 3 confirm that in all farm types, except for the extensive farms in the UK, the sale of lambs for meat is the predominant activity, contributing on average by more than 65% in their gross revenue (ranges from 65.6% to 69.94%). In the UK, apart from the sale of lambs sold for meat which accounts for 38.74% of gross revenue, the sale of lambs for breeding constitutes the predominant activity, contributing by 43.74% in their gross revenue. An interesting finding is that in all cases the share of the value of lambs sold for meat is higher in the relatively inefficient farms than the efficient. Moreover, the results show that the sales of lambs for breeding is an important source of income in all types of efficient farms, indicating it as an important way of adding value to the enterprise. 

The share of coupled subsidies in gross revenues varied from 13.32% in the intensive French farms to 26.91% in the Spanish farms, indicating that sheep farms rely heavily on public support and are vulnerable to policy changes; a finding which implies that these farms must improve their competitiveness. It must be mentioned that no data were available on subsidies for the UK farms. Moreover, it should be noted that in the case of French farms, both intensive and extensive, coupled subsidies represented a higher percent of gross revenues in the inefficient compared with the efficient group. These results are in accordance with a previous meta-analysis involving studies across the agricultural sector, which reported a negative association between subsidies and TE of farms [39]. The share of the value of culled animals was, as expected, very high in the UK (14.67%), where they are used to consuming heavy carcasses and varied from 1.48% in Spain to 5.48% in France. Regarding the UK, it should be noted that the share of culled animals on the gross revenue was much higher in the efficient group (21.19%) compared with the inefficient (12.63), indicating the economic benefits from the exploitation of their meat. Contribution of wool in gross revenue was trivial, ranging from 2.85% in the UK to 4.05% in Spain, and its value did not vary significantly between the efficiency groups.

The main technical and economic data of the two efficiency groups as well as of the average farm were compared, providing a description of the structure and the profitability of the farms that utilize efficiently the production technology (Table 4). The comparison of the main technical and economic characteristics between the average efficient and the relatively inefficient farm partly indicated their contribution to the improvement of the level of TE.

Results showed that in all farm types, the largest flocks were reared by the efficient farms, which produce more lamb meat per ewe in kg. The only exception was the UK, where the efficient farms were mainly selling lambs for breeding and not for meat. Concerning lamb meat, it has to be clarified that the data originally provided by Spain and the UK reported lamb carcass meat sold off-farm. The conversion to the number of lambs sold was based on estimations regarding carcass weight per lamb provided by experts from each country. Moreover, in all cases, the efficient farms appeared to manage more rationally human labor, using less labor per ewe. The latter finding can be attributed to the fact that they are larger in size, benefiting from economies of scale. Efficient farms utilized from 512 ewes per Annual Labor Unit (ALU) in French extensive farms to 1246 ewes per ALU in the UK. Furthermore, efficient farms, apart from the intensive farms in France, supplied less feed per ewe, a finding that suggests a higher dependency of these efficient extensive and semi-intensive farms on grazing and/or an excessive use of feed by the inefficient farms. In the intensive farm type, efficient farms supplied more dry matter per ewe (DM supplied is 27 kg per ewe higher in the efficient farms) as expected, since in the intensive farms, lambs are confined and fattened indoors.

Labor cost, in most cases, followed the trend in labor use, thus, it was reduced in the efficient French and UK extensive farms by 14 EUR/ewe and 11 EUR/ewe, respectively, whereas in the intensive French and semi-intensive Spanish farms there was no differentiation between the efficiency groups. Feeding cost was reduced by 5 EUR/ewe in the efficient extensive French and UK farms; however, it was increased by 6 EUR/ewe in the efficient intensive French farms. An interesting finding is that the extensive efficient farms seem to depend more on home-grown feed, whereas the intensive and semi-intensive farms depend more on purchased feed. Sheep farmers even under the same production system apply different feeding strategies; some produce home-grown feed, whereas others prefer to purchase a large part of their feed from markets and a debate regarding which strategy is the most profitable differ [29,40]. Another interesting result is that the fixed cost, which is the most important source of production cost followed by feeding cost, was much higher in the inefficient farms. This result indicates that inefficient farms are characterized by irrational and excessive investments on fixed capital (mainly buildings and machinery) and/or poor capital management. This is further supported by a previous study in Spanish sheep meat farms, which also reported important investments on installations not proportional to the farms’ size and needs [23]. Moreover, this finding can be partially attributed to the fact that efficient farms, which rear large flocks, operate under a more intensive pattern, decreasing their fixed cost per animal by using their infrastructure at full capacity. Only in the Spanish farm type the fixed cost per ewe did not differentiate among efficiency groups. The lowest production cost occurred in the case of the efficient farms for all farm types except for the Spanish farms, where there was no variation of total cost between the efficiency groups. Furthermore, the highest gross revenue was also achieved by the efficient farms in all cases (gross revenue varied from EUR 157 per ewe in the Spanish farms to EUR 214 per ewe in the intensive French farms), indicating that a higher level of efficiency is related to a higher value of production. Consequently, gross margin (gross revenue less the variable cost), was increased in the efficient farms and varied from EUR 82 per ewe in the UK extensive farms to EUR 125 per ewe in the intensive French farms. The indicator of net profit (or loss) shows that in the long term, both types of Spanish farms and the efficient farms in the UK will be economically viable.

Efficient farmers indicated the management and production practices that are implemented on their farms in the most effective way or at least better than in the other farms, having a major impact on farms’ sustainability and economic performance. These practices are implemented at full potential on the efficient farms and constitute the practices that could be potentially introduced to farms of same or similar production system as innovations. This feedback obtained from the farms was analyzed providing valuable information regarding best practices. These practices are classified into nine general categories for each farm type in France and Spain in Table 5. There was no feedback available from the UK farms. More specifically, Table 5 presents the number of the efficient farmers that selected at least one farm practice from a specific general category (4th column of Table), and the type of practice that was selected at least once by an efficient farmer (last column). The categories of practices are presented in descending order, according to the number of efficient farms that selected the best practices.

Feeding practices were indicated by efficient farmers of all types as those that make their farms operate better than the others. Specifically, 16 of the 25 efficient French extensive farmers (64%), 17 of the 22 efficient French intensive farmers (77%) and 6 of the 14 efficient Spanish semi-intensive farmers (42.8%) selected at least 1 feeding practice from 6 different types of practices. These practices were related, among others, to feed budgeting, i.e., good understanding of matching animal requirements and supply, increased forage and pasture quality, use of additives, innovative grazing practices and use of by-products to replace conventional feeds. Various breeding practices that included the use of elite flocks, system/criteria to choose best animals for replacement, production data collection (i.e., milk yield/quality), DNA data collection and use in breeding programs and new traits to increase resilience and efficiency were implemented by 15 extensive (60%), 14 intensive (63.6%) and 5 semi-intensive (35.7%) farmers. 

Implementation of modern technology through gadgets and applications was also pointed out as a practice that could promote solutions at farm level by 13 extensive (52%), 18 intensive (81.8%) and 6 semi-intensive (42.8%) farmers. The types of practices that were selected by the farmers included electronic identification systems, on-farm data collection linked to animal ID and feedback to farmer to help decision making, temporary electric fencing in mountainous areas, GPS control and automatic animal handling. Practices that are associated with product marketing (such as product certification, branding and provenance of products for more local and direct markets, better use of the environmental and social aspects of sheep farming in the marketing of sheep meat, attractive branding, greater differentiation of products) were also highlighted by a large share of the efficient farmers (48% of the extensive, 77.3% of the intensive and 42.8% of the semi-intensive farmers). On the contrary, practices related to product processing were not indicated as important for the performance of the farms, presumably because it mainly concerns the processing sector.

Many farmers also reported that access to information and farmer’s training is essential to improve the sector’s sustainability and efficiency. Specifically, 10 extensive (40%), 17 intensive (77.3%) and 2 semi-intensive (14.3%) consider that access to abattoir feedback on carcass quality and health, use of farm management software and training on breeding programs are among the practices that make them better than their peers. Moreover, as it was expected, reproduction practices were also deemed as salient features of the efficient operation of the farms. Among the efficient farms, 12 extensive (48%), 13 intensive (59.1%) and 6 semi-intensive farms (42.9%) reported that assisted reproduction techniques improved use of rams, frequently reviewed reproduction plans and improved fertility through better quality of frozen semen, which are key factors for a profitable sheep meat production. 

Practices that are related to a better organization of human resources, namely staff training courses and regular meetings to obtain feedback and keep positive stimulation, as well as monitorization of labor cost/use, were also reported by 10 extensive (40%), 6 intensive (27.3%) and 6 semi-intensive farms (42.8%) as practices that distinguish them from other farmers. On the contrary, animal health management practices that included use of antibiotic alternatives in feeding and identification tests to spot animals with illness were selected by few farmers as practices that ameliorate their farms’ performance. 

Overall, results showed that in all farm types, feeding, breeding and reproduction practices, farmers training programs, modern technology applications as well as product marketing practices were highly implemented by the efficient farmers and were pointed out as the features that distinguish them from their peers. These best-observed practices could potentially constitute the innovations that should be undertaken by other farms to enhance their efficiency and productivity. 

Undoubtedly, the adoption of such innovations and practices must be tailored to specific conditions, i.e., farm types, production systems, climatic area, etc. Based on the analytical results, differences were observed between countries and farming systems regarding the prioritization of best practices/innovation categories. This can be attributed to the diverse technical, financial and environmental/social challenges that each production system faces [16]. In this regard, gadgets and apps are more likely to be adopted in intensive, large-sized farms with skilled workers and high investments in facilities, whereas feeding strategies utilize pastures in extensive, low-input farms. Therefore, innovation strategies should be in accordance with the farm type’s specific features and challenges and adjusted for the needs of a wider territorial strategy. 

Further analysis within the general, “broad” categories of practices revealed those that are mostly pointed out by the efficient farms as best practices/innovations (Table 6). Most of the efficient farms (49 of the 61 farms, i.e., 80% of the total efficient farms) suggested the use of electronic identification systems. The use of electronic identifiers (ear tags and/or boluses) is mandatory for sheep farmers within the EU, since it is an efficient way of ensuring traceability of animals and their products. Moreover, the use of electronic identification readers facilitates animal performance recording, and hence genetic improvement [14], as well as animal health recording and prevention of diseases [41]. However, on-farm data collection linked to animal ID for decision making was indicated as best-practice by 30% of the farms (Table 6). Utilization of electronic identification systems for animal monitoring and data collection in extensive sheep meat production systems is hampered by the high rate of ear tag loss as well as by economic constraints. Therefore, initiatives for a simplified identification system combined with financial support for extensive sheep meat farmers are imperative [14]. Furthermore, training farmers on the usefulness of electronic identification systems and strengthening their capability in use of technology could increase their uptake [41,42]. 

Feed efficiency is also at the center of farmers’ concerns due to the high costs of purchased feed and the volatility of prices that jeopardize farms’ sustainability. In this context, innovations on feeding practices involve mainly the development of optimal feeding strategies that increase self-sufficiency, the efficient utilization of pastures and forages and the provision of alternative feeds [43]. In this study, the most observed best-practice in feeding was the understanding of animal requirements and matching those with feed supply (69% of farms, Table 6). Increased forage quality, innovative grazing practices and increased pasture quality were also indicated by many farms (48%, 38% and 36%, respectively) as practices that ameliorate the farm’s performance. Finally, 15% of the farms suggest the use of by-products to replace conventional feeds. In sheep meat farms feeding cost accounts for 60–70% of the total variable cost. Therefore, such innovative practices to efficiently utilize pastures and replace conventional feeds, whose cost is continuously increasing, with plant by-products could contribute to the improvement of the economic performance of farms [44]. Previous research has also shown that the use of such by-products could have favorable effects on meat quality [45,46,47]. The latter combined with the positive environmental impact through reduction in feed transportation and ruminant CH4 emissions allows for new product marketing opportunities [45,46,47]. Hence, the use of alternative feeds, such as by-products from the agri-food industry, should be encouraged to decrease the risks associated with climate change and feed price volatility and ensure a fair income for the farmers.

There is also considerable need for innovation in small ruminant products and marketing strategies, especially when comparing with other livestock sectors. New packaging and cuts, development of quality labels and traceability systems and new marketing campaigns are key strategies for the development of the sector [48,49]. In the present analysis, 64% of the efficient farms indicated that certified meat products are crucial for the competitiveness of the sector, whereas 20% of the farms focused on the branding of their products for more local and direct markets (Table 6). According to previous research with Spanish, French and UK consumers [50,51], one of the most important attributes when selecting lamb meat is its local origin and certification such as Protected Designation of Origin (PDO) and Protected Geographical Indication (PGI). For such products with known local identity consumers are willing to pay higher prices, supporting, therefore, a sustainable economic and social development of the sector. Generally, in the EU, sheep and goat farms that are oriented towards the production and sale of meat face profitability problems, not mainly due to increasing operations costs but due to low sales prices for meat. Relevant campaigns could further assist to increase the attractiveness of such products and society’s awareness [14].

Amongst breeding practices observed in the efficient farms, the use of elite flocks, following a specific system or criteria to choose the best animals for replacement and practicing routine data collection, were selected by 62%, 60% and 54% of farms, respectively (Table 6), as innovations that make them better than their peers. Accurate pedigree information and collection of on-farm performance data of individual animals are essential for accurate genetic selection [52]. Moreover, using high breeding value rams from elite flocks ensures efficiency and speeds up the genetic improvement process [53]. Nowadays, breeding innovations are associated mainly to the advances in molecular genetics and DNA analysis, which have boosted the development of new tools in breeding programs and to the formation of selection indexes which combine productional and functional traits [54,55,56]. However, such innovations were suggested by only 20% of the studied farms (Table 6). To increase the uptake of breeding innovations (genetic and genomic) and their efficient implementation, specific breeding goals should be set and well-organized; the long-term collaboration of farmers, breeding associations, scientists and government structures needs to be promoted [52].

Likewise, assisted reproduction technologies and the use of improved rams and reproduction plans were pointed out by 56% and 44%, respectively, as practices that improve animals’ performance and consequently farms’ profitability. Artificial insemination is known to increase the selection intensity and the precision of genetic evaluation, therefore enhancing the efficiency of genetic improvement. Specifically, according to previous research in French sheep meat farms, an increase in genetic gain of 57% to 84% can be achieved with the use of artificial insemination when combined with progeny testing [57]. Moreover, it minimizes the need of rams and hence, is economically beneficial for the farmer. However, even in the case of natural mating, the use of improved rams ensures genetic improvement and production optimization.

Regarding information and training, access to abattoir feedback on carcass quality and health and the use of farm management software programs were indicated by 44% and 23% of the efficient farms, respectively (Table 6), as practices that improve farm’s performance. Evaluation of live sheep as well as their carcasses in abattoirs can provide useful insight regarding health and welfare on farm. Farmers can utilize this feedback for efficient health management planning and welfare-related corrections for better meat quality [58]. In terms of computer-assisted farm management there are plenty of tools available. Specifically, there are tools for sustainability assessments at the farm level [59,60], for individual animal recording of production and health aspects [16,61] and for decision making through predictive modeling [62]. Such tools can provide farmers with economic, environmental and social indicators, assist breeding programs and/or help with efficient farm management planning for higher production and profits [62]. In order to increase the adoption of such tools, training of farmers and consultancy are imperative [63].

In general, farmers’ training is the key to improving the sector’s overall sustainability, and working towards this direction is essential to maximize animal performance and farm’s profitability. In the present study, staff training courses were pointed out by 31% of the efficient farms as crucial for the improvement of the sector’s productivity (Table 6). Initiatives to promote farmer interaction with animal husbandry professionals in order to increase knowledge and improve expertise and participatory farmer group training programs appear to be a strategy with high potential to develop a more knowledgeable and competent farming workforce [16,55]. However, national organizations with regional branches to oversee the implementation of such programs, a strong network of all stakeholders and reliable funding sources are required [55].

Finally, in terms of animal health, 15% of the efficient farms indicated the use of antibiotic alternatives in feeding (Table 6) as a practice, which distinguishes them from the inefficient ones. The consistent use of antibiotics in feed has been found to promote antibiotic-resistant microorganisms. Hence, natural alternatives such as tannins and essential oils that have antimicrobial properties could be utilized in small ruminant nutrition [64,65]. Health-related best practices were greatly underrepresented in the present study. This suggests that special emphasis should be given on educating farmers on relevant best practices that could help towards efficient health management planning. 

## 4. Conclusions

A major transition has emerged in the European sheep sector in the last two decades and sheep meat farmers are continuously facing challenges that require adaptation and innovation to keep farms competitive. In order for the sheep sector to remain fully integrated into the liberalized market, a shift to more intensive patterns and more innovation and technology are required. The improvement of farm technology, as well as boosting innovations in farm practices, processes and products, is a strategic priority for the small ruminant sector in general [66]. 

In this study, efficiency analysis proved to be a practical tool for identifying best practices, innovations and management strategies that will help sheep farms to achieve resilience and increase profitability. The efficiency analysis revealed high variation in both extensive and intensive sheep farms, indicating that there is potential to increase their output if they utilize properly existing production technologies. Results showed that more efficient meat farms comprise large flocks, use their infrastructure at full capacity reducing fixed cost and manage human labor rationally.

Moreover, farmers indicated which production and management practices are considered crucial for the competitiveness and the profitability of the sector, and should be adopted as innovations by other farms. Only farms which take up innovative solutions to modernize and rationalize their modus operandi are likely to remain in business with an emphasis on increasing flock size to utilize economies of scale, management of feeding and grazing and marketing strategies. Moreover, breeding programs that include genomic information and current technological trends such as digital technologies, Internet of Things and decision support tools must be used to re-design and support the sheep sector. 

However, this study indicates that innovations in sheep farming should be designed and implemented in a trans-disciplinary, multi-stakeholder and participatory context. The successful implementation of such innovative practices in the small ruminant sector is prevented from socioeconomic and structural constraints both at the farm and sector level. Therefore, any research/extension service program or action promoting innovations on sheep farming should firstly aim to overcome these constraints that hinder innovation uptake.

## Figures and Tables

**Table 1:** Frequency distribution of farms by technical efficiency (TE) estimates from the DEA model.

Efficiency	French Extensive Farms			French Intensive Farms			Spanish Semi-Intensive Farms			UK Extensive Farms		
	ΤΕ			ΤΕ			ΤΕ			ΤΕ		
	No of Farms	%	Mean	No of Farms	%	Mean	No of Farms	%	Mean	No of Farms	%	Mean
<0.60	66	27.38	0.496	1	1.64	0.576	2	5.41	0.580	18	15.1	0.528
0.60–0.69	52	21.58	0.649	7	11.48	0.655	3	8.11	0.647	15	12.6	0.647
0.70–0.79	47	19.50	0.749	11	18.03	0.753	3	8.11	0.744	37	31.1	0.744
0.80–0.89	41	17.02	0.844	12	19.67	0.851	10	27.03	0.848	19	16.0	0.837
0.90–0.99	10	4.15	0.953	8	13.11	0.951	5	13.51	0.909	8	6.7	0.927
1.00	25	10.37	1.000	22	36.07	1.000	14	37.83	1.000	22	18.5	1.000
Total	241	100.0	0.709	61	100.0	0.873	37	100.0	0.874	119	100.0	0.773

**Table 2:** Average existing and efficient frontier output for sheep farms by farm size.

	Farm Categories			Average Farm
	Small	Medium	Large	
French extensive farm				
No of farms	80	45	116	241
Existing output (Gross revenue in EUR)	37,751	63,741	118,818	81,624
Efficient target (Gross revenue in EUR)	57,088	97,980	155,235	111,964
French intensive farm				
No of farms	22	13	26	61
Existing output (Gross revenue in EUR)	51,439	83,262	125,254	91,582
Efficient target (Gross revenue in EUR)	55,119	102,512	138,373	104,035
Spanish semi-intensive farm				
No of farms	13	13	11	37
Existing output (Gross revenue in EUR)	80,270	97,990	219,504	127,890
Efficient target (Gross revenue in EUR)	87,343	121,109	239,840	144,543
UK extensive farm				
No of farms	44	46	29	119
Existing output (Gross revenue in EUR)	38,069	84,524	221,813	100,805
Efficient target (Gross revenue in EUR)	50,571	115,986	269,104	129,113

**Table 3:** Composition of gross revenue per ewe for the technical efficient and inefficient farms.

Efficiency Groups		Composition of Gross Revenue (EUR/ewe)					
		Lambs Sold For Meat	Lambs Sold for Breeding	Cull Animals	Compensations-Subsidies	Wool and Other Products	Total
French Extensive Meat Farm							
Inefficient (TE ^1^ = 0.675)	Mean	101.58	12.79	6.77	27.39	4.51	153.04
	%	66.38	8.36	4.42	17.89	2.95	100.0
Efficient (TE ^1^ = 1.000)	Mean	109.25	30.37	8.02	26.35	4.57	178.56
	%	61.18	17.01	4.49	14.76	2.56	100.00
Average farm (TE = 0.709)	Mean	102.64	15.22	6.94	27.25	4.52	156.57
	%	65.56	9.72	4.43	17.4	2.89	100.0
French Intensive Meat Farm							
Inefficient (TE ^1^ = 0.802)	Mean	125.81	9.94	10.78	25.25	5.77	177.55
	%	70.86	5.59	6.07	14.22	3.26	100.0
Efficient (TE ^1^ = 1.000)	Mean	147.44	25.39	10.06	25.93	5.81	214.63
	%	68.69	11.83	4.69	12.08	2.71	100.0
Average farm (TE = 0.873)	Mean	134.01	15.79	10.50	25.51	5.79	191.6
	%	69.94	8.24	5.48	13.32	3.02	100.0
Spanish Semi-Intensive Meat Farm							
Inefficient (TE ^1^ = 0.798)	Mean	92.75	0.69	2.19	35.47	3.91	135.01
	%	68.69	0.52	1.62	26.28	2.89	100.0
Efficient (TE ^1^ = 1.000)	Mean	100.28	2.87	2.06	43.47	8.45	157.13
	%	63.82	1.83	1.31	27.66	5.38	100.0
Average farm (TE = 0.874)	Mean	95.95	1.61	2.13	38.86	5.85	144.4
	%	66.48	1.11	1.48	26.91	4.05	100.0
UK Extensive Meat Farm							
Inefficient (TE ^1^ = 0.722)	Mean	57.81	60.44	17.61	NA	3.60	139.46
	%	41.45	43.34	12.63	-	2.58	100.0
Efficient (TE ^1^ = 1.000)	Mean	42.66	63.91	30.06	NA	5.25	141.88
	%	30.07	45.04	21.19	-	3.70	100.0
Average farm (TE = 0.773)	Mean	54.24	61.26	20.55	NA	3.99	140.04
	%	38.74	43.74	14.67	-	2.85	100.00

^1^ TE = technical efficiency.

**Table 4:** Technical and economic characteristics of the technical efficient (E) and inefficient (I) farms.

Technical and Economic Data	French Meat Extensive			French Meat Intensive			Spanish Semi-Intensive Meat			UK Extensive Meat		
	Efficiency Groups		AF ^1^ (TE ^2^ = 0.709)	Efficiency Groups		AF ^1^ (TE ^2^ = 0.873)	Efficiency Groups		AF ^1^ (TE ^2^ = 0.874)	Efficiency Groups		AF ^1^ (TE ^2^ = 0.773)
	I (TE ^2^ = 0.675)	E (TE ^2^ = 1.000)		I (TE ^2^ = 0.802)	E (TE ^2^ = 1.000)		I (TE ^2^ = 0.798)	E (TE ^2^ = 1.000)		I (TE ^2^ = 0.722)	E (TE ^2^ = 1.000)	
Technical												
Number of farms	216	25	241	39	22	61	23	14	37	97	22	119
Number of ewes	501	693	521	464	502	477	829	1003	894	675	918	720
Lambs sold (per farm)	432	621	452	471	588	513	1278	1651	1419	460	428	454
Lambs sold (per ewe)	0.86	0.90	0.87	1.01	1.17	1.07	1.54	1.65	1.58	0.68	0.48	0.63
Total labor (ewes/ALU)	385	512	399	517	520	518	519	551	532	853	1246	922
Feed supplied (Kg DM/ewe)	390	307	378	486	513	496	308	257	287	93.55	62.58	86.24
Economic												
Labor cost (EUR/ewe)	68	54	66	52	51	51	31	31	31	35	24	32
Feed cost (EUR/ewe)	45	40	45	57	63	60	59	60	59	20	15	19
Purchased feed (EUR/ewe)	34	31	34	42	48	45	41	45	42	13	7	12
Home-grown feed (EUR/ewe)	11	9	11	15	15	15	18	15	17	7	8	7
Variable Capital cost ^3^ (EUR/ewe)	25	30	26	28	26	27	9	7	9	47	45	46
Fixed Capital cost (EUR/ewe)	81	66	78	79	71	76	22	23	23	68	48	64
Production cost (EUR/ewe)	219	190	215	216	211	214	121	121	121	170	131	161
Gross revenue (EUR/ewe)	153	178	157	178	214	192	135	157	144	140	142	140
Gross margin (EUR/ewe)	83	108	86	93	125	105	67	90	77	73	82	75
Profit or Loss (EUR/ewe)	−66	−18	−58	−38	3	−22	14	36	23	−30	11	−21

^1^ AF = average farm. ^2^ TE = technical efficiency. ^3^ Includes veterinary and drug expenses, expenses for bedding and animal recording, and land rent (only for the UK).

**Table 5:** General categories of best observed practices and innovations in efficient French and Spanish farms.

General Category of Practices	Country	Farm Type	No of Eff Farmers that Selected at Least One Practice	Types of Practices
Feeding	France	Extensive	16/25	6
	France	Intensive	17/22	6
	Spain	Semi-intensive	6/14	5
Breeding	France	Extensive	15/25	4
	France	Intensive	14/22	6
	Spain	Semi-intensive	5/14	3
Gadgets and Applications	France	Extensive	13/25	3
	France	Intensive	18/22	6
	Spain	Semi-intensive	6/14	3
Product marketing	France	Extensive	12/25	6
	France	Intensive	17/22	5
	Spain	Semi-intensive	6/14	3
Information and training	France	Extensive	10/25	5
	France	Intensive	17/22	4
	Spain	Semi-intensive	2/14	2
Reproduction	France	Extensive	12/25	3
	France	Intensive	13/22	3
	Spain	Semi-intensive	6/14	2
Human resources organization	France	Extensive meat	10/25	2
	France	Intensive	6/22	2
	Spain	Semi-intensive	6/14	2
Health	France	Extensive	8/25	4
	France	Intensive	3/22	2
	Spain	Semi-intensive	2/14	1
Product processing	France	Extensive	2/25	2
	France	Intensive	2/22	2
	Spain	Semi-intensive	0/14	0

**Table 6:** Most observed best practices and innovations across farm types.

General Categories of Practices	Practices	No of Farms
Gadgets and Apps	Electronic identification systems	49/61
Feeding	Understanding of matching animal requirements and supply	42/61
Product Marketing	Certification of products	39/61
Breeding	Use of elite flocks	38/61
Breeding	System/criteria to choose best animals for replacement	37/61
Reproduction	Assisted reproduction techniques	34/61
Breeding	Routine data collection (i.e., milk yield/quality)	33/61
Feeding	Increased Forage Quality	29/61
Reproduction	Improved rams and reproduction plans	27/61
Information and Training	Access to abattoir feedback on carcass quality and health	27/61
Feeding	Innovative Grazing Practices	23/61
Feeding	Increased Pasture Quality	22/61
Human resources Organization	Staff training courses/regular meetings to get feedback	19/61
Gadgets and Apps	On-farm data collection linked to animal ID for decision making	19/61
Information and Training	Computer farm management programs	14/61
Breeding	DNA data collection and use in programs	12/61
Human resources Organization	Monitorization of labour costs/efficiency	12/61
Product Marketing	Branding of products for more local and direct markets	12/61
Feeding	Use of by-products to replace conventional feeds	9/61
Health	Scientific proven use of antibiotic alternatives in feeding	9/61

## Data Availability

The data presented in this study are available in Supplementary Materials (Spreadsheets S1–S4 and Template S1).

## Supplementary Materials

The following are available online at https://www.mdpi.com/article/10.3390/ani11113242/s1, Spreadsheet S1: Technical and economic data collected from extensive sheep meat farms in France, Spreadsheet S2: Technical and economic data collected from intensive sheep meat Farms in France, Spreadsheet S3: Technical and economic data collected from semi-intensive sheep meat farms in Spain, Spreadsheet S4: Technical and economic data collected from extensive sheep meat farms in the United Kingdom, Template S1: Template for innovations and best observed management practices used to record the feedback of most efficient farms.
