# Supplementary material for: Efficiency Analysis as a Tool for Revealing Best Practices and Innovations: The Case of the Sheep Meat Sector in Europe
Source: Animals (Basel). 2021 Nov 12;11(11):3242. doi: 10.3390/ani11113242 (PMC8614382; doi:10.3390/ani11113242)
Supplement: Supplementary file 1 [file animals-11-03242-s001.zip › Template S1.pdf]

Template for Innovations and best observed management practices

**Innovative and best observed management and production practices**	**Please, indicate with an "X" in the relevant row which are the practices that efficient farms apply the best/at full potential**	**Description**
**A. Farm Management**		
A.1 Feeding		
- Innovative Grazing Practices	X	e.g. "Implementation of high-throughput feed evaluation techniques"<br>e.g. "Set of criteria to evaluate grass state to decision making about rotating grazing plots"
- Increased Pasture Quality		e.g. "Use of legumes and high sugar grass"
- Increased Forage Quality		
- Use of by-products to replace conventional feeds		
- Good understanding of matching animal requirements and supply (feed budgeting)	X	e.g. "Grouping animals with similar requirements"
- Use of additives	X	-
- Other		
A.2 Health		

- Sound and scientific proven use of antibiotic alternatives in feeding		
- Identification tests to spot animals with illness		
- Use regionally integrated plans		
- Use of sensor RFID ear-tags as welfare indicators		
- Cortisol Hair analysis		
- Other		
A.3 Reproduction		
- Improved fertility through better quality of frozen semen		
- Assisted reproduction techniques		
- Improved/frequently reviewed of use of rams and reproduction plans		
- Other		
A.4 Breeding		
- Routine data collection (i.e. milk yield/quality)		
- Use of elite flocks		

- System/criteria on place to choose best animals for replacement		
- New traits to increase resilience and hardiness		
- DNA data collection and use in programs		
- Other		
A.5. Human resources organization		
- Monitorization of labour costs/efficiency		
- Staff training courses / regular meetings to get feedback and keep positive stimulation		
B. Farm Technology		
B.1 Information and Training		
- Integrated and easy-to-use tools		
- Access to abattoir feedback on carcass quality and health		
- Tools to monitor BCS and pasture state		
- Computer farm management programs		

- Training on maximizing breeding programs and resources		
- Other		
B.2 Gadgets/ Apps		
- GPS control		
- Drones		
- Temporary electric fencing in mountainous areas		
- Electric identification systems		
- On-farm data collection linked to animal ID and feedback to farmer to help decision making		
- Automatic animal handling		
- Animal stress automatic sensors		
- App to collect animal welfare indicators		
- Electronic microchip readers and automatic milk recording systems for individual milk production		
- Other		
**C. Product Processing and Marketing**		

C.1 Product Processing		
- Low fat / Omega 3 enriched products		
- Freeze drying for longer storage and exportation to China		
- Goats milk		
- Vegetable rennet		
- New products		
- New smarter packaging		
- New cuts / products		
- Innovation to increase halal slaughtering		
- High standard animal welfare measures to improve quality of product at processing		
- Stress free slaughter for improved meat quality		
- Other		
C.2 Product Marketing		
- Promote fresh sheep and goat products		

- Better use of the environmental and social aspects of sheep farming in the marketing of sheep meat		
- Branding and provenance of products for more local and direct markets		
- Attractive branding, greater differentiation of product		
- Certification		
- Other		
